# The Ambiguity of *Artworks* –A Guideline for Empirical Aesthetics Research with Artworks as Stimuli

**DOI:** 10.3389/fpsyg.2017.01857

**Published:** 2017-10-24

**Authors:** Gregor U. Hayn-Leichsenring

**Affiliations:** ^1^Psychology of Beauty Group, Institute of Anatomy I, University Hospital Jena, Jena, Germany; ^2^Department of Neurology, University of Pennsylvania, Philadelphia, PA, United States

**Keywords:** art, artwork, definition, intension, theoretical

## Abstract

The aim of this work is to provide researchers from the field of aesthetics with a guideline on working with artworks as stimuli. Empirical aesthetics research is complicated by the uncertainty of the object of research. There is no way to unquestionably tell whether an object is an artwork or not. However, although the extension of the term artwork (i.e., the range of objects to which this concept applies) remains vague, the different intensions of the term artwork (i.e., the internal concept that constitutes a formal definition) are well defined. Here, I review the various concepts of artworks (i.e., intensions) that scientists from different fields use in current research in empirical aesthetics. The selection of stimuli is often not explained and/or does not match the focus of the study. An application of two or more intensions within one study leads to an indeterminacy of the stimuli and, thus, to systematic problems concerning the interpretation and comparability of the experimental results. Based on these intensions and the Pleasure-Interest Model of Aesthetic Liking (Graf and Landwehr, 2015), I compiled a decision tree in order to provide researchers with an instrument that allows a better control over their stimuli.

## Introduction

‘Art is indefinable.’ This familiar sentence of unknown origin seems to be a central tenet of current aesthetics research. According to Weitz (1956), there is no property that is common to all types of art. Of course, scientists in the field of aesthetics are aware of the complex problems that arise from the uncertainty of their research object. Several models of aesthetics research have been established (Leder et al., 2004; Jacobsen, 2006; Chatterjee and Vartanian, 2014; Graf and Landwehr, 2015; Redies, 2015). These models are, however, not focused on artworks as stimuli, but on particular aspects of aesthetic experience. Therefore, the models do not reflect the use of artworks as stimuli in experiments, but they rather replicate the perception and processing of the artwork or of other stimuli during experiments. This is a critical point. Presumably, in every scientific model in the field of aesthetics, the focus is on the participant (observer or artist) in the experiments, while the experimental decisions taken by the respective researcher (e.g., on the stimuli used, or on the purpose of the conducted experiment) are largely ignored. These decisions, however, are crucial for the interpretability of the results. Like every scientific field, aesthetics research that uses artworks as stimuli requires rules in order to avoid arbitrariness. I will argue that scientific research in the field of aesthetics would strongly benefit from being aware of the ontological state of the artworks, the applied intensions of the term artwork and the mode of processing that is investigated. Furthermore, I propose a decision tree that makes it easier to select stimuli for their experiments, to make decisions with regard to the acquisition of participants and to avoid some common research problems. The present text focuses mostly on art paintings. However, similar conclusions might be drawn for other types of artworks as well.

## Approaches to the Term Artwork

First of all, a definition of the investigated object is of utmost importance for every scientific experiment. There are two major issues that complicate a definition of the term *artwork*: (1) The ontological state of artworks and (2) the differentiation between extension and intension for this term. In the following, these issues will be analyzed.

### The Ontological State of Artworks

**FIGURE 1:**
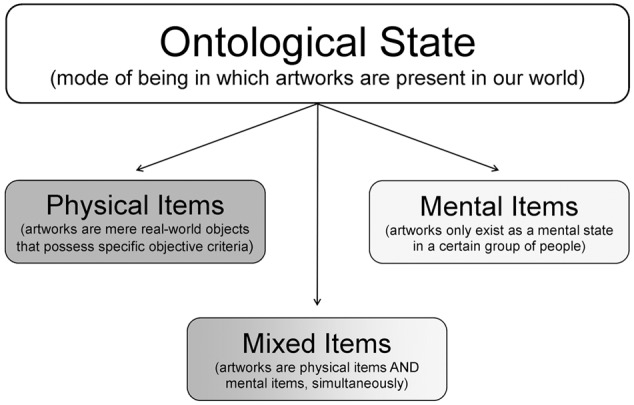
The ontological state of artworks. If artworks are physical items, they are real-world objects and there must be objective criteria in order to add items to this group. If artworks are mental items, they only exist as a mental state in a certain group of people. In this case, the only reason for a physical object to be denoted as an artwork is the mode of perception or the attitude of the observer toward the respective item. Due to the complex character of artworks, it seems impossible to settle for one of the two options. Therefore, a third option has been introduced (Schmücker, 2003). Artworks are mixed items, i.e., they are physical items and mental items, simultaneously.

The ontological state of *artworks* denotes the mode of being in which artworks are present in our world. Ontologically, it is possible to describe *artworks* as *physical items* or as *mental items* (**Figure 1**). However, both of these notions do not sufficiently characterize *artworks*. Therefore, it is necessary to describe *artworks* as *mixed items* meaning that they are, in part, physical items and, in part, mental items (**Figure 1**). Following this notion, Schmücker (2003) denotes artworks as *intersubjective-instantial type entities* (German translation: “intersubjektiv-instantiale Typ-Entitäten”). This term can be defined as follows: Artworks are entities of a specific class (or type) of items that exist as mental states in a group of people (intersubjective) and, simultaneously, have (or have had) a physical representation in the real world (instantial). In order to refer to an item as *artwork*, three essential conditions must be fulfilled: (1) There has to be a specific far-ranging consensus on the item that classifies it as artwork, (2) there is or there was at least one incident that led to its creation and (3) there is at least one physical manifestation – or there is at least one mental state that guarantees the possibility of its physical manifestation (Schmücker, 2003). The ambiguity of the ontological state is reflected in the intensions of the term *artwork*.

### The Extension and the Intension of the Term Artwork

Since the term *artwork* escapes a universal definition, the only possibility to define this term is to take a differentiated (philosophical) look at it. Here, it is helpful to discriminate between the *extension* and the *intension* of artworks. *Extension* means that a term is defined by examples. In other words, the term is defined by the range of items to which the term applies (Carnap, 1947). For instance, the extension of the term *car* consists of all actual cars (BMW 318i, Hyundai i30, etc.). In the case of the term *artwork*, the extension approach is to great extent related to the assumption that ‘Art is indefinable’ and, therefore, can only be described by examples. Obviously, the extension of the term *artwork* is not very useful if one wants to define what the essence of an artwork is. Instead, its essence can be described with the *intension* of the term. *Intension* implies that a term is defined by its inner meaning. The intension relates to the properties that an object needs to have in order to be counted as referential to the term. In other words, an intensional definition provides the meaning of a term by specifying necessary and sufficient conditions for when the term should be applied. Therefore, the intension aims at the internal concept that constitutes a formal definition. Referring to the example term *car*, the intension would be that a car (1) is a self-powered motor vehicle that (2) is used for transport, (3) is a product of the automotive industry, (4) runs primarily on roads, (5) has seating for one to eight people and (6) has four wheels. These six intensions of the term *car* apply to all or at least most cars. This is the case for most non-philosophical terms.

*Artwork*, however, is an ambiguous term. For centuries, philosophers and scientists from different fields argued over the different intensions of the term *artwork* (Kant, 1790; Zimmermann, 1865; Bell, 1914; Adorno, 1970; Tatarkiewicz, 1972; Danto, 1981; Gaut, 2005). The intensions can be paraphrased as certain points of view on a specific item (i.e., artwork) and include essentialism, production-aesthetic intentionalism and reception-aesthetic intentionalism, mental functionalism, historicism, institutionalism and the cluster account. Here, it is not the aim to argue for one specific intension, to investigate the intensions in detail or to pinpoint their advantages and disadvantages. Instead, I will focuses on the problems that arise in scientific research if two or more intensions of the investigated item are applied in a mixed fashion. The central issue is that – in contrast to *cars* – artwork cannot be described with every intension of the term.

In everyday life, it is not problematic to apply more than one intension of the term *artwork* to one specific item. When somebody visits an art gallery, the classification of an object as artwork is not of practical relevance. In scientific research, however, the application of more than one intension within one study is highly problematic because an exact interpretation of results is impossible without control over the experiment. This is true for the selection and usage of the stimuli in particular.

## Intensions of the Term Artwork

In nearly every scientific study, researchers have to make two choices: (1) Which items are used in the study? (2) Which aspects of the items are investigated? For most (non-aesthetic) experiments, the selection and usage of items go hand in hand. Sticking to the example *car*, one could apply the criterion “objects that are self-powered” for selection and investigate “transport” facilities. This is easily possible because both intensions can be applied simultaneously. However, if the investigated items are denoted with ambiguous terms (e.g., *artwork*), this is not the case. In order to explain this problem in more depth, the different intensions of the term *artwork* will be briefly introduced, following the description by Piecha (2003). Due to the nature of scientific articles, the descriptions have to be quite short and rather incomplete. Nevertheless, they will convey the quintessence of the respective intension.

### Essentialism

**FIGURE 2:**
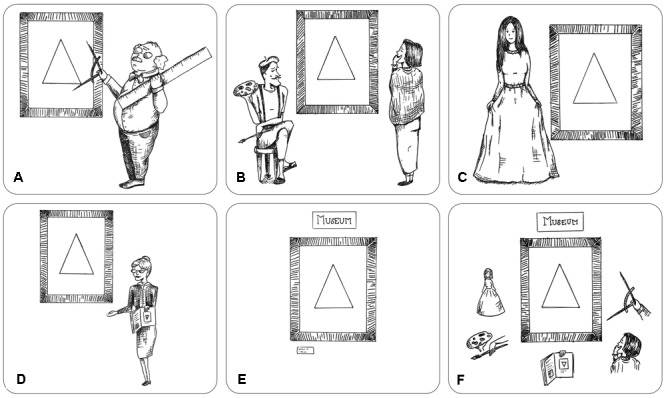
Graphic display of five intensions of the term artwork. **(A)** Essentialism; **(B)** Intentionalism; **(C)** Functionalism; **(D)** Historicism; **(E)** Institutionalism; **(F)** Cluster Account. Drawings by Nathalie Lyssenko.

Following the conviction that artworks are physical objects, they must possess a materialistic medium within our world. This assumption leads to the essentialistic approach (see **Figure 2A**). The main statement of the essentialism (or formal objectivism) is that there are formal (perceivable) object properties that are universal to any artwork (Fechner, 1876; Bell, 1914; Osborne, 1952; Redies, 2015). Most essentialistic hypotheses aim at *beauty* and not at the decision whether an object is an actual artwork. At least until the late 19th century, art had been closely associated with beauty (Tatarkiewicz, 1972). Nowadays, several research groups focus on formal aspects of beauty and found, for instance, preferences for a specific ratio of organization divided by complexity (Birkhoff, 1933), for curved over sharp objects (Bar and Neta, 2006), for symmetry (Jacobsen et al., 2006), and for self-similarity (Redies et al., 2012). However, preferences for certain object properties cannot define whether an item is an artwork or not. The complex ontological state of artworks – they are no simple physical objects, but also have an inherent mental component – opposes this assumption. Nevertheless, universal properties of artworks are widely discussed.

### Intentionalism

In contrast to essentialism, in which artworks are considered as purely physical objects, intentionalism also takes into account mental aspects, that is, artworks are considered as mixed items. In this view, artworks are man-made items that transmit emotions and/or other non-verbal information (see **Figure 2B**). Usually, the (supposed) intention of the artist is considered to be detached from an influence on the perceiver. Therefore, intentionalism can be divided into two types: (1) The production-aesthetic intentionalism and (2) the reception-aesthetic intentionalism. In the production-aesthetic intentionalism, the artist‘s intention is communicated through the artwork. Danto (1981) argues that the intention of the artist to produce an artwork and to decide what is expressed in this particular artwork is of utmost importance. According to him, it is impossible to perceive an artwork without an interpretation of it. This credo also applies to the reception-aesthetic intentionalism in which an artwork is an item that has an influence on the perceiver. In visual aesthetics, participants’ reactions to artworks are frequently measured (Augustin et al., 2011; Leder et al., 2013). With the application of reception-aesthetic intentionalism, an investigation of inter-individual differences between participants is possible as well. In this body of research, personality traits have been linked to preference differences for art styles (Furnham and Walker, 2001) and specific types of abstract artworks (Lyssenko et al., 2016).

### Functionalism

The mental functionalism also considers artworks as mixed items. To some extent, mental functionalism is related to the reception-aesthetic intentionalism. The main difference is that functionalism is not focusing on individual perceivers, but rather on general functions of artworks. From a functionalistic point of view, an artwork is a social construct that has to fulfill a specific purpose (e.g., as a symbol, see **Figure 2C**). The term *artwork*, therefore, applies to items that represent something. For a visual artwork to be a symbol, it does not necessarily have to resemble an original item in order to function as iconic representation (Goodman, 1968). Instead, it can also *exemplify* particular properties. Pale colors, for instance, can represent sadness or loneliness. Therefore, exemplification is a kind of interpretation – but an interpretation that can be followed quite easily. Partly in line with this, Adorno (1970) proposes a dialectic approach, according to which every critically relevant artwork criticizes society and is the source of utopian power. Therefore, the artwork – and Adorno emphasizes especially the form, not the content – relates to history and societal relationships. According to him, artworks illustrate the state of the society and, in particular, its decadence.

### Historicism

Historicism can be seen as a special version of functionalism. In historicism – in contrast to Adorno’s assumptions – items get the status of artworks only if they relate to previous artworks (see **Figure 2D**) – and not necessarily to society, as it is the case in functionalism. Carroll (1999) claims that we identify an item as an artwork if it is possible to embed the item in a contingent story in relation to previous items that have already been identified as artworks. Though artworks are described as items that show whatsoever similarity to other artworks (at least for most of the topics), the mental reflection about these items is of equal importance. Similar to intentionalism and functionalism, artworks are seen simultaneously as physical objects and as mental items (mixed items) in historicism.

### Institutionalism

The actual physical form of appearance (or manifestation) of an artwork is completely negligible in institutionalism (see **Figure 2E**). For institutionalists, an artwork is an item that has been nominated as an artwork. Dickie (1974) proposed that an item is an artwork (1) if it is a candidate for appreciation and (2) if it is presented to the *art world* by authorized representatives. In this scenario, the institutionalism itself functions as a kind of black box, in which some item is put and, as a result, there is an evaluation whether it is an artwork or not (Piecha, 2003). This process can be rather arbitrary. It can, however, also be based on some of the other intensions mentioned above because the representatives (or “experts”) might evaluate items according to essentialistic, intentionalistic, historic or functionalistic criteria. While this is certainly the case for some artworks, the actual process of evaluating the artwork often remains rather obscure in most cases.

### Cluster Account

The cluster account as proposed by Gaut (2005) combines all previously presented intensions (see **Figure 2F**). Following it, there is no generally favorable concept of the term *artwork*. Instead, items can gain the status of artworks by exhibiting certain physical properties by their communicating intentions, their influence on the perceiver, their symbolic function, their relation to art history, their simple nomination or by a combination of these intensions. Supporters of the cluster account declare that it is impossible to categorize every approved artwork (following the extension of the term) by one of the mentioned intensions. After an item has been denoted as artwork, the justification for this denotation is investigated. A major problem is the *post hoc* characteristic of this approach. By identifying an item as an artwork, it automatically receives a new state in the world. A subsequent interpretation, as proposed by supporters of the cluster account, necessarily depends on this newly acquired state (Piecha, 2003).

## The Two Processing Modes

Researchers performing experiments with artworks as stimuli should not only be aware of the previously described aspects of the stimulus (ontological state and intensions). When performing experiments with participants, they should also decide which mode of processing of the stimulus is investigated.

A major portion of empirical aesthetics research focuses on underlying mechanisms of aesthetic perception. Here, the *fluency* of a given stimulus has been identified as an essential feature (Reber et al., 1998, 2004a). Fluency is thought to facilitate processing and, therefore, increase aesthetic liking.

**FIGURE 3:**
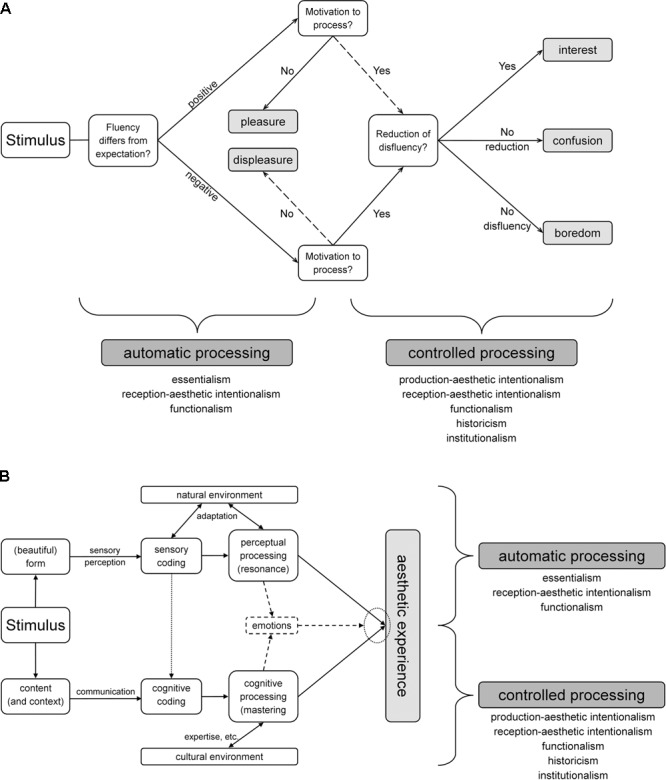
The processing modes of aesthetic stimuli. Displayed are two models in which the two processing modes *automatic processing* and *controlled processing* are hierarchical **(A)** or simultaneous **(B)**. **(A)** The Pleasure-Interest Model of Aesthetic Liking (PIA Model), modified after Graf and Landwehr (2015). The PIA Model claims that a discrepancy between expected and experienced fluency leads to affection (pleasure or displeasure, *automatic processing*). Afterward, if the perceiver senses the need for cognitive enrichment and explores the item, a reduction of disfluency can lead to an enhanced interest (*controlled processing*), while the absence of reduction leads to confusion and the absence of disfluency leads to boredom. See section “The Two Processing Modes” for further details. **(B)** The Model of Aesthetic Experience (AE Model), modified and simplified after Redies (2015). The author differentiates between perceptual processing (resembling *automatic processing*) and *cognitive processing* (resembling *controlled processing*). Both ways of processing take place at the same time and are partly intertwined. See section “The Two Processing Modes” and the original article for further details.

In the Pleasure-Interest Model of Aesthetic Liking (PIA Model), Graf and Landwehr (2015) differentiate two kinds of fluency, the so-called *automatic processing* and the so-called *controlled processing* [**Figure 3A**, analogously, e.g., Jakesch et al. (2013) differentiate between *perceptual fluency* and *cognitive fluency*]. Automatic processing involves the processing of presumably fluent objective properties that have been associated with aesthetic liking. These are, for example, symmetry (Wurtz et al., 2008), clarity/contrast (Reber et al., 1998, 2004b) and fractality (Taylor et al., 1999; Redies, 2007). Additionally, the perceivers’ experience has been taken into account. With this regard, repeated exposure/familiarity (Bornstein and Dagostino, 1994; Furnham and Walker, 2001), duration of exposure (Reber et al., 1998) and perceptual priming (Reber et al., 1998) have been identified to increase aesthetic liking as well. Automatic processing is mandatory, unintentional and it is mainly stimulus driven (Berlyne, 1974; Graf and Landwehr, 2015). Therefore, it can be associated with essentialism [perceivers’ reaction to objective properties of artworks; Hayn-Leichsenring et al. (2017)], reception-aesthetic intentionalism (artworks’ direct influence on the perceiver, e.g., evoking emotional responses) and functionalism [perceivers’ direct reaction on exemplifications based on previous experiences; Belke et al. (2015)].

In contrast, controlled processing occurs if a stimulus receives sufficient intention by the observer. It is perceiver driven and requires an active and reflective interaction with the stimulus (Graf and Landwehr, 2015). For instance, appreciation of car interiors (Carbon et al., 2013) and exterior designs (Landwehr et al., 2013) can be affected by asking participants to evaluate the stimuli on different dimensions. Furthermore, information on titles (Millis, 2001; Leder et al., 2006) and style (Russell, 2003; Belke et al., 2006) can enhance the appreciation of artworks showing that if the elaboration is meaningful, the liking of artworks is increased. Additionally, research on insight claims that active elaboration of the perceiver leads to a higher aesthetic interest (Muth and Carbon, 2013). Controlled processing can be associated with production-aesthetic intentionalism (perceivers’ attempt to understand the artists’ aim), reception-aesthetic intentionalism [e.g., insight; Muth and Carbon (2013)], functionalism [e.g., cognitive understanding of the function of the artwork; Bullot and Reber (2013)], historicism [integration of the artwork in the art world; Bullot and Reber (2013)] and institutionalism [e.g., influence of museum context; (Brieber et al., 2015)].

The authors of the PIA Model claim that automatic processing and controlled processing are hierarchical (Graf and Landwehr, 2015). In contrast, the Model of Aesthetic Experience (AE Model) by Redies (2015) proposes a simultaneous occurrence of the two processing modes (**Figure 3B**). It is not the aim of this article to decide whether the processing modes are hierarchical or simultaneous. Instead, they are linked to different intensions of the artwork. This means that in order to conduct adequate experiments, researchers should decide whether they investigate automatic or controlled processing.

## Artworks as Stimuli in Aesthetics Research

Next, I will introduce four different research problems emerging from the ontological state of artworks and the application of different intensions within one study. As stated before, every researcher usually has to make two choices: (1) Which items are used in the study? (2) Which aspects of the items are investigated?

(1) The first choice aims at the selection of the stimuli. The question is: Which factors trigger the selection process for the stimuli? Consciously or subconsciously, researchers decide which items are referred to as artworks and, subsequently, are used as stimuli in the study. Most of the time, scientific researchers assume that the only (and unquestioned) criterion for an item to be considered as an artwork is that it is or was displayed in a museum. However, the selection is then left to art historians or art curators who follow a selection process that is not sufficiently explained. Superficially, this approach seems to be a selection according to institutionalism. However, as described in section “Institutionalism”, the selection process of the experts might be based on other intensions. Therefore, there is often no good control over the stimuli used in experiments (see The Incalculable-Cluster-Account Problem).
(2) The second choice aims at the usage of the stimuli. In some of the studies in the field of aesthetics, it is not clearly stated which intension of the term *artwork* is targeted in the investigation. In order to point out the purpose of the study, researchers should reveal how the stimuli are treated and which aspects of them are important.

If the first choice and the second choice do not match (i.e., the aim of the study has not been adequately taken into account when selecting the items that are used in the study) problems may occur. In order to explain the necessity of stimulus awareness, four of these problems associated with the handling of the stimuli will be described and analyzed in the following sections. Two of those examples focus on the ontological state of artworks (perception-mode problem and mixed-items problem) and two examples focus on the intensions of the term *artwork* (two-intensions problem and incalculable-cluster-account problem). In order to illustrate these points, I will provide some examples. By no means, however, I intend to disqualify the studies that are discussed below. Instead, some of the interpretations of the results will be discussed. All of the selected studies have been conducted by rightfully renowned researchers who contributed greatly to recent empirical aesthetics research. I selected the studies randomly having in mind that a large portion of empirical aesthetics research on artworks suffers from similar problems. In order to describe the occurring problems, however, a detailed explanation of the studies is required because, in most cases, these problems might not be obvious to readers. To increase readability, only a small number of studies have been included.

### The Perception-Mode Problem

Referring to their ontological state, artworks are mixed items (Schmücker, 2003). If an object is perceived as an artwork, it possesses a specific inherent mental component and, therefore, the perception mode differs between artworks and non-artworks. This issue has been addressed in a study on disgusting objects. When items were claimed to be artistic, affective responses by the participants were more positive (Wagner et al., 2014). Furthermore, labeling IAPS (International Affective Picture System) images as artworks reduces positive emotional reactions (Gerger et al., 2014). In other words, we look differently at an object when we classify it as an artwork. This might be based on the expectation of deeper meaning, which may lead to a more intensive look at the object (Danto, 1981). For this reason, inferring from experiments on general perception to perception of art, and vice versa, is highly problematic. Of course, it would be unfair to say that one can never draw conclusions on general perception from results that stem from experiments, in which artworks are used as stimuli. But for all that, researchers should be very careful when assigning conclusions from mixed items to materialistic items.

As stated before, artworks (mixed items) are perceived in a way different from non-artworks (materialistic items). Marin and Leder (2016) showed that complexity ratings after long exposure time differed between photographs of environmental scenes and representational art paintings. Possibly, cognitive interference in art perception (controlled processing) that is related to associations and interpretation attempts might influence the judgments (Marin and Leder, 2016). Furthermore, several studies showed that the confrontation with artworks can activate the so-called default mode network (a network of interacting brain regions that is usually active when the focus is not on the outside world). Interestingly, the activation of this network is mediated by different artworks in different participants (Vessel et al., 2012, 2013). The default mode network is not activated when participants perceive non-artistic stimuli (materialistic items). Therefore, if researchers want to draw conclusions on art perception from experiments on non-art stimuli (e.g., Muth and Carbon, 2013), an elaborate discussion is inevitable. Only then, it might be possible to consider a transfer of the results.

A related problem occurs when researchers alter images of artworks. In a study on ambiguity, Jakesch et al. (2013) investigated paintings by René Magritte. They used two different versions of 36 Magritte paintings: an original version and a photoshopped version, in which the inherent ambiguity had been removed. The authors stated that “the original and the manipulated version only differed in terms of ambiguity […]” (Jakesch et al., 2013, p. 5). However, this statement is not entirely correct. The images did not only differ in terms of ambiguity but also in terms of their ontological state. Consequently, the authors compared ambiguous images of artworks (i.e., mixed items) with non-ambiguous images that were no artworks (i.e., materialistic items). A similar problem can be found in several other studies (McManus et al., 1993; Di Dio et al., 2011). It has been shown that digital images that are thought to be created by the researcher are less appealing than digital reproductions of artworks (Kirk et al., 2009). Most importantly, images that have been artificially created by scientist are – in most cases – not works of art. Though they are solely generated to fulfill a specific purpose (i.e., to function as stimuli in an experiment), they do not qualify by any intension to be categorized as artworks. An exception is the reception-aesthetic intentionalism. Possibly, participants perceive these images as works of art. However, if this is assumed, the researcher has to control for participants’ perception mode. One possibility is to ask participants *post hoc* if they think they saw artworks as stimuli and, therefore, control for *perceived authenticity* (Pelowski et al., 2017). Perceived authenticity influences the evaluation of a painting’s quality as well as the artist’s talent (Wolz and Carbon, 2014). While asking participants whether the perceived stimuli were images of artworks is surely a helpful approach, even if participants state that they got the impression to be confronted with artworks, this does not solve the problem entirely. If the participant perceives a non-art image and – based on his assumption that the image depicts a real artwork – reacts falsely *as if* this is an image of an artwork, it is not plausible to expect the exact same reaction as if the participant perceives the image of a real artwork. A naïve viewer may be not affected by certain features of artworks (no automatic processing of fluency) but at the same time categorize the stimulus as artwork because of the expectation to be confronted with art and/or his misleading assumptions how art should look like. Nevertheless, a *post hoc* report by the participants whether they assume that they perceived images of real artworks could be helpful to control the results. In any case, the results of the respective experiments should be interpreted carefully. In other words, researchers should be aware of the specific aesthetic perception mode for artworks.

### The Mixed-Items Problem

Another problem based on the ontological state of artworks is the mixed-item problem. Researchers often treat artworks as physical items and neglect their mental component. However, if artworks were simple physical objects, a (perfect) copy of the artwork would be indistinguishable from the original. This is, however, not the case. Therefore, showing pictures of artworks on a computer screen is – in many cases – highly problematic. While it is possible to measure image properties (a perfect copy possesses presumably the same image properties as the original), it is implausible, for instance, to study certain reception-aesthetic intentionalistic effects with these copies. As the same physical reaction are not elicited when we see a photograph of Anne Hathaway or Hugh Jackman than when we meet them for real, it is also not the same to see a picture of an artwork and to be faced with the original. In a study on titles of abstract artworks, for instance, Leder et al. (2006, p. 181) presented computer images of abstract artworks to participants, as it is commonly done in experiments. Then, they asked whether the participants “understood the artist’s intention,” “whether the artwork affected them emotionally,” and what their “thoughts evoked by the artwork” were. Of course, the participants answered these questions. But, in fact, it was illegitimate to ask such questions because no real artworks were present. The aura of the original and, subsequently, the mental state of the respective item makes artworks special. Therefore, representations of at least some artworks will not evoke the same effect in the observer as the originals do. Similar problems can be found in other studies (Vartanian and Goel, 2004; Villani et al., 2015).

The effect of representations of artworks is rather difficult to examine. One has to differentiate between the already mentioned *perceived authenticity* (Pelowski et al., 2017) and the *actual authenticity*. Even if authenticity is correctly perceived – meaning that the participant that is confronted with an image of a real artwork reacts *as if* he is confronted with the real artwork– a copy does not feature the aura and the temporal and spatial uniqueness of the original (Benjamin, 1969). Therefore, the reaction to the copy might be different (Locher et al., 1999). In summary, researchers should be aware that some investigations are illegitimate when performed on mere copies of actual artworks (for details, see section “Control over Stimuli”). Thus, such experiments should be avoided or they should be conducted with actual artworks.

A rather similar problem arises from the location of the experiment. Researchers should be aware of the difference between a museum setting and a laboratory setting. Usually, visitors are in a certain state of mind when visiting a museum (Brieber et al., 2015) which might be related to the prestige of institutionalism in our time (Pelowski et al., 2017). When participating in an experiment, their state of mind is not necessarily the same. For instance, the contour of the room influences ratings on beauty and pleasantness (Vartanian et al., 2013). Additionally, other aspects of presentation context like framing (Redies and Gross, 2013; Ensor and Hamilton, 2014), boundaries (Cupchik, 2006), lighting (Griswold et al., 2013) and size (Pelowski and Akiba, 2011) have been discussed to be relevant for art appreciation. Quite often studies on artworks target on the state of mind of the perceiver. In these cases, the location of the participant has to be considered in order to reach a valid interpretation of the results (Cela-Conde et al., 2011; Gartus and Leder, 2014).

### The Two-Intensions Problem

The application of two different intensions for the same items/stimuli in scientific research can lead to erroneous conclusions. This discrepancy becomes problematic, for example, if the selection and the experimental usage of the stimuli do not hold up to the same standards. Two examples of hypothetical cases will demonstrate this issue. Afterward, three actual studies will be described.

#### The Obvious Example: Measuring a Black Square

**FIGURE 4:**
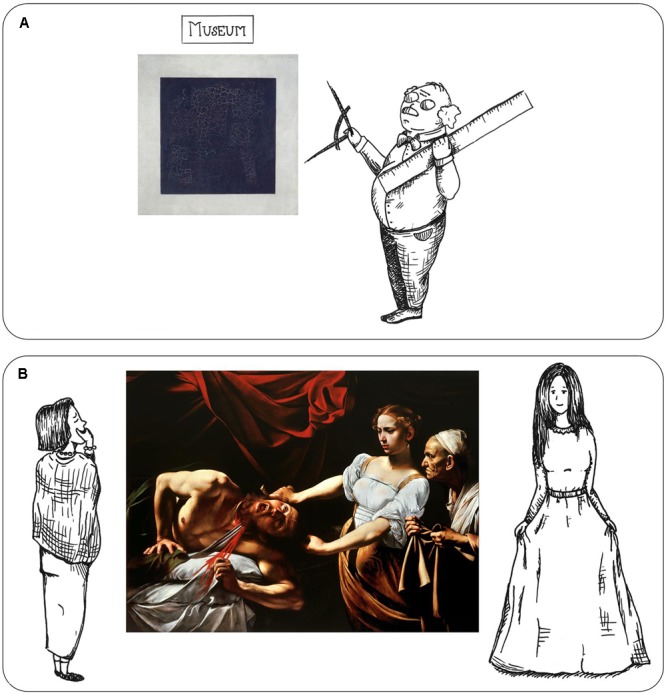
Graphic display of problems stemming from the application of two different intensions in aesthetics research. **(A)** See section “The Obvious Example: Measuring a Black Square” and **(B)** see section “The Less Apparent Example: Bible Knowledge and Caravaggio”. Drawings by Nathalie Lyssenko.

The first example deals with a rather obvious discrepancy (see **Figure 4A**). Let us assume that Researcher 1 plans an experiment on the formal aspects of artworks (essentialism, automatic processing). He found a new method to measure a certain image property and wants to test whether this property is universal in artworks. He selects his stimuli from different museums from all around the globe, also from the Tretyakov Gallery in Moscow. One of the stimuli is the painting “Black Square” (1915) by Kazimir Malevich. Malevich, however, painted “Black Square” with the intention to escape form and color, and to create some kind of anti-artwork. He stated: “(Black Square is meant to evoke) the experience of pure non-objectivity in the white emptiness of a liberated nothing” (Drutt, 2003). For this reason, the selection of the stimulus to be displayed at Tretyakov Gallery is presumably based on production-aesthetic intentionalism (Malevich created an item whose classification as artwork is mainly based on mental aspects). This kind of art mainly aims at controlled processing. Therefore, it is improper to measure the physical size or structure of “Black Square” and expect compelling results. Of course, no conscientious researcher would make this mistake. The inaccuracy is way too obvious in this case. However, when the application of the intensions is hidden, it gets more complicated.

#### The Less Apparent Example: Bible Knowledge and Caravaggio

The second example deals with a more sophisticated situation (see **Figure 4B**). Researcher 2 plans an experiment on the physical reaction of observers that are confronted with cruelty in artworks (reception-aesthetic intentionalism). He uses Baroque paintings with gruesome and bloody displays as stimuli. One of the selected paintings is “Judith Beheading Holofernes” (1599) by Caravaggio. This painting shows a horrible scene, in which a young woman cuts off the head of an older man. Some (naïve) observers might therefore react with disgust, fear or horror (automatic processing within reception-aesthetic intentionalism, as, possibly, expected by Researcher 2). However, other observers who are well versed in the Bible and art history may recognize the painting as a depiction of the story of Judith and Holofernes (automatic and controlled processing within functionalism). In this story, Judith, a beautiful widow, decapitates the drunken and lustful Holofernes in order to save her hometown. The whole story – and, subsequently, the painting – is an established symbol for lust as weakness of men (as part of the “Power of Women” *topos*). In this case, participants who are familiar with the symbolism may react differently, because in comparison to art experts, lay people tend to refer to personal feelings as criteria to evaluate artworks (Augustin and Leder, 2006). If Researcher 2 is not aware of this factor, the results lack validity because the possible knowledge of the inherent symbolism by some participants may have a major influence on their reception.

#### Three Examples from Actual Research

In the following, I will discuss three actual studies. Two of them will serve as examples for the two-intensions problem, while the third is an example for well-crafted and adequate research. Again, I emphasize that the criticized studies were selected randomly and that similar problems occur in several other studies. In the interest of readability and conciseness, I will focus on very few examples.

The study by Chen et al. (2008) is an example for problems that can occur if stimuli are not deliberately chosen. They used three images as stimuli: “The Starry Night” (Vincent van Gogh), “Mona Lisa” (Leonardo da Vinci) and an anonymous landscape photograph. The stimuli were introduced as “Three pieces of art” (Chen et al., 2008) although there was no evidence that the landscape photograph can be considered as an artwork. Furthermore, the authors did not state why they chose these diverse stimuli. Supposedly, the selection process for the van Gogh painting and the da Vinci painting was partly based on their fame (institutionalism). However, the use of the landscape photograph as stimulus remained obscure. The authors drew general conclusions on the appreciation of artworks by schizophrenic people (reception-aesthetic intentionalism). However, as described before, the reaction to a small reproduction of an artwork and the reaction to the original artwork at its original size will be different (see also section “The Perception-Mode Problem“). Furthermore, “The Starry Night” and “Mona Lisa” differ so much in their content that they are not equivalent for the purpose of the experiment. While “The Starry Night” is an emotional rollercoaster with a wild and turbulent sky contrasted by a calm town, “Mona Lisa” is an example for a beautiful portrait of an elegant and attractive woman without visual distractions. Therefore, the physical and psychic reactions to these paintings, let alone reproductions of these paintings, are necessarily rather different and cannot be measured on the same scale. If researchers want to take such differences into account, they could perform a pre-study to identify reproductions of art paintings that evoke similar emotions in perceivers (see section “Control over Participants”). Besides, there are two other problems with the study: (1) It is impossible to infer from a study on two artworks to general conclusions on art appreciation. (2) The two chosen art paintings are very famous and, therefore, will be perceived and evaluated in different manner than the photograph.

In a study on orientation of artworks, Mather (2012) selected forty stimuli according to the following four criteria: “(i) there was a clear intended or “correct” orientation, (ii) the work was produced by an artist of international renown, and therefore of high aesthetic quality, (iii) the set as a whole represented a sample of modern art from the early to mid-20th century, and (iv) paintings varied in the extent, to which they exhibited recognizably representational content.” (Mather, 2012, p. 19). Obviously, the author followed institutional and (rather vague) production-aesthetic intentional intensions for the selection of stimuli. Participants had to decide which orientation of the artwork was the “most pleasing or meaningful” (Mather, 2012, p. 19) to them (reception-aesthetic intentionalism). Again, there is a problem with the study design: The intensions for selection and usage of the stimuli were not the same. For some painters, the orientation might not have mattered at all – or they might have oriented the images differently, due to the smaller size of the reproductions used in the experiment presented (resizing images modifies the visual impression). Nevertheless, the author still drew conclusions on the actual artwork: “In the case of Kasimir Malevich’s Suprematist Composition, 72% of responses selected an incorrect orientation rotated 90° anticlockwise from the correct orientation” (Mather, 2012, p. 23). However, only information on how participants oriented the resized images is provided whereas the choice that the artist would have taken under the same experimental conditions remains elusive. It is thus problematic to draw conclusions on any art-related matter in such an experiment. In order to avoid this problem, the researcher should have used stimuli for which the artists explicitly stated the “correct” orientation (production-aesthetic intentionalism) – at the best even for the smaller representations of the artworks. There is another problem with this study: Paintings are not necessarily aesthetically pleasing only because the artist is famous (reception-aesthetic intentionalistic intension).

In contrast to the two previously presented studies, Augustin et al. (2011) showed a better sensibility for their stimuli in an EEG study on the timeline of processing style and content of artworks. In their study, “stimuli were reproductions of 50 paintings by the French Post-Impressionist Paul Cézanne (1839 – 1906) and 50 paintings by the German Expressionist Ernst Ludwig Kirchner (1880 – 1938) who represented the two levels of the style dimension in the materials” (Augustin et al., 2011, p. 2074). The authors chose the stimuli based on a pre-study that legitimated the categorization of the stimuli and trained the participants in artistic style. In this case, the selection and the usage of stimuli were based on a reception-aesthetic intentionalistic intension. Therefore, this study serves as a good example for an adequate research in empirical aesthetics.

Researchers should choose their stimuli according to the purpose of the study and – in order to produce *internally valid* (within one study) and *externally valid* (between studies) results – they should carefully evaluate which intension they applied for the selection and usage of the stimuli. The final article should not only include a statement on the usage of the stimuli (aim of the study), but also the selection of the stimuli should be described in detail.

### The Incalculable-Cluster-Account Problem

In many cases, researchers select their stimuli based on institutionalism. As described above, the selection process of museums could or could not be associated with another intension. If a researcher selects classic and modern artworks based on institutionalism (i.e., they are displayed in a museum), he ignores the fact that the choice of the museum to display them may have been based on various factors. Classic artworks are subject to a different selection process than modern artworks. On the one side, the selection of classic artworks is, presumably, often based on essentialistic and production-aesthetic intentionalistic intensions. On the other side, modern artworks are, presumably, often chosen because of historic, functionalistic and reception-aesthetic intentionalistic intensions. The key point is that researchers are probably not aware of the selection process in most cases. This problem can be denoted as the incalculable cluster account. Every stimulus selection based on institutionalism is problematic.

In an eye movement study on visual interest, Locher et al. (2007) used images of eight paintings by renowned artists from The Metropolitan Museum of Art, New York. The stimuli “were selected to represent a range of styles along the abstract-representational continuum” (Locher et al., 2007, p. 57). As already pointed out above, the selection process for museum paintings is often rather obscure. One could assume that Jan Vermeer’s “Young Woman with a Water Pitcher” (1660–1662) was chosen for its artistic beauty (essentialism) while Fernand Léger’s “Woman with a Cat” (1921) was chosen as a prime example of cubism (historicism). Alternatively, it is conceivable that the paintings were exhibited in the museums just because they were works by famous artists. The point is that we do not know what led to their exhibition. It cannot be ruled out that such decisions have an influence on the results of the study. Several other studies face similar problems (Mallon et al., 2014; van Paasschen et al., 2014; Villani et al., 2015). In contrast to the first three problems, there is no solution for this problem. Researcher in the field of aesthetics should avoid the incalculable-cluster-account problem and not base their stimulus selection on institutionalism.

## A Guideline for Empirical Aesthetics Research

**FIGURE 5:**
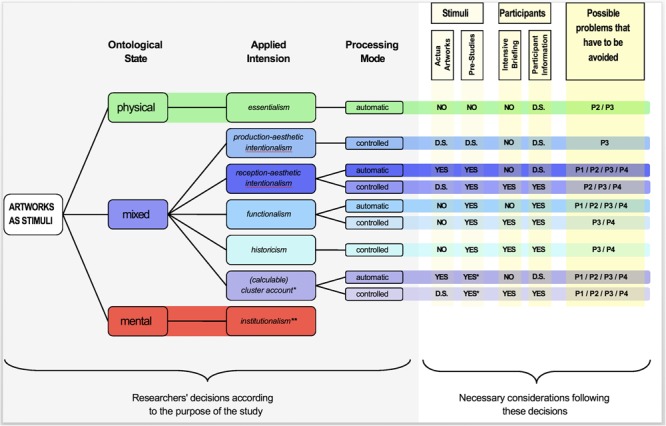
Decision tree for working with artworks as stimuli. Displayed is the way of decisions (from left to right) a researcher has to take when conducting a study on artworks. First, the researcher has to decide which ontological state of the artwork is considered. This decision does not have to reflect the view of the researcher, but rather the way artworks (i.e., stimuli) are treated in the respective study. For instance, although very few researchers vote for artworks as purely materialistic objects, several studies focus on objective properties. The second decision is on the applied intension of the term artwork. I encourage researchers to become aware of the different intensions and recognize their respective notion (see **Figure 2**). Especially in science, it is of utmost importance to define the item of interest. It is not sufficient to refer to the statement ‘Art is indefinable.’ Instead, researchers should specify which intension of the term artwork is investigated in their specific study (this decision relates to the purpose of the study and not to the selection of the stimuli). After this specification, the model of aesthetic appreciation and aesthetic judgments (Leder et al., 2004) provides researchers with several options for specific aspects of aesthetics research (on artworks). For example, the ‘Perceptual Analysis’ in the model is closely related to essentialism, while ‘Cognitive Mastering’ refers to production-aesthetic and reception-aesthetic intentionalism. The third decision aims at the question which processing mode will be studied. As previously described, the PIA Model (Graf and Landwehr, 2015, see **Figure 3A**) as well as the AE Model (Redies, 2015, see **Figure 3B**) differentiate between two processing modes (automatic and controlled) which should be investigated separately. Following these three decisions, different aspects concerning the stimuli, participants and possible research problems have to be considered. For a detailed description of the consequences of the decisions for control over stimuli, control over participants and possible research problems, see sections “Control over Stimuli”, “Control over Participants”, and “Awareness for Possible Research Problems”. YES = required; NO = not required; D.S. = depending on the specific study. P1 = Perception-Mode Problem, P2 = Mixed-Items Problem, P3 = Two-Intensions Problem, P4 = Incalculable-Cluster-Account Problem. Please see sections of similar names in the manuscript for a detailed description of each possible Problem. ^∗^In a calculable cluster account, the researcher consciously applies different intensions in order to investigate differences between them. Usually, at least two pre-studies should be conducted to create varying sub-datasets of stimuli that have been chosen based on one particular intension. ^∗∗^As mentioned in the text, the application of institutionalism for the selection of stimuli necessarily leads to an Incalculable-Cluster-Account-Problem (section “The Incalculable-Cluster-Account-Problem”) and, for this reason, should be avoided.

In this review, I argued that research on artworks is highly complicated because of the special ontological state, the manifestations of non-compatible intensions and different processing modes. There is no simple way to determine which kind of precautions have to be taken and what mistakes should be avoided in an aesthetic study. These considerations are highly dependent on the approach taken, on the aim of the study and on specific circumstances. Nevertheless, I provide a guideline in form of a decision tree (**Figure 5**) in order to give an overview of the decisions that a researcher has to make before he starts an experiment and to show the consequences of those decisions. In research with artworks as stimuli, three decisions have to be made: (1) On the ontological state of the artworks, (2) on the applied intension of the term *artwork* for the purpose of the stimuli and (3) on the investigated processing mode (see **Figure 5** for further details). These decisions are highly important for considerations on stimuli control, handling of the participants, as well as for the awareness for the possible occurrence of research problems (as described in sections “The Perception-Mode Problem,” “The Mixed-Items Problem,” “The Two-Intensions Problem,” “The Incalculable-Cluster-Account Problem”).

### Control over Stimuli

Researchers should be aware that the selection of the stimuli has an effect on the results. While this principle is basically true for every kind of research, it becomes even more important in aesthetics research because the stimuli are denoted with an ambiguous philosophical term (*artwork*). In order to produce internally valid (within study) results, the researcher has to select his stimuli according to the purpose of the study.

First, the respective researcher has to decide whether it is required to use actual artworks as stimuli. In general, the usage of images (copies) of artworks is problematic if reactions of participants are measured (reception-aesthetic intentionalism and cluster account, automatic processing). When focusing on the mere physical aspects of the artwork (essentialism), it is sufficient to use copies. Also, studies investigating functionalistic and historistic aspects can be carried out with copies because it is not necessary to see the original artwork in order to understand its symbolism and/or to file it into the art world. In other cases, the respective usage has to be challenged.

Secondly, researchers should decide whether pre-studies for the selection of stimuli are necessary. Pre-studies are especially useful if artworks are considered as mixed items. They are helpful to gain a better control over the stimuli and to match them to the purpose of the study. Concerning stimuli, pre-studies should be used as a tool to create a dataset that is adequate to the aim of the study. For instance, artworks for the pre-study could be selected based on the institutionalism and the pre-study itself filters out the particular artworks that are symbols with a specific purpose (functionalism). Thus, pre-studies can be used to convert an incalculable cluster account to an intension of choice (Augustin et al., 2011). Of course, it is not always required to apply the same intension of the term artwork to the selection of stimuli and their investigation. Instead, every singular study has to be challenged. If the purpose of the experiment is to investigate the difference between two or more intensions (calculable cluster account), several pre-studies should be carried out in order to create according datasets.

### Control over Participants

Due to the subjectivity of aesthetic experiences, the control over participants is of utmost importance in aesthetics research. Especially when investigating controlled processing, an intensive briefing of the participants (providing participants with information on the stimuli/experiment, leading the focus of the participant, etc.) is necessary because controlled processing largely depends on the state of mind of the participant. In contrast, automatic processing should be largely unaffected by briefing (Graf and Landwehr, 2015).

Additionally, some studies require the acquisition of information on the participants beyond usual data (e.g., gender, age, and educational degree). For instance, degree of art expertise, familiarity with the stimuli, religious beliefs and mood could be of interest. This information is essential in experiments on controlled processing because of the influence of personal characteristics. Also if the application of functionalism is the purpose of the study, the study will benefit from information on the participants. Some functional aspects are processed automatically (e.g., subconscious reaction on colors as representatives for certain moods). This might also be the case for investigations of essentialistic and intentionalistic characteristics. Therefore, prior to these studies, the acquisition of information on the participants should be considered.

### Awareness for Possible Research Problems

One purpose of the decision tree is to display, which researcher decisions may lead to what kind of problems. The possible problems have been elaborated in section “Artworks As Stimuli in Aesthetics Research”. Most of these problems can be avoided if researchers are aware of the challenges that come with their decisions. The decision tree (**Figure 5**) gives an overview on which problems are likely to occur in which experiments.

Additionally, I strongly encourage researchers to state the intensions that have been applied. In order to conduct externally valid (between studies) experiments, researchers should not only state which intension of the term *artwork* they focus on in their investigation (usage of stimuli, see **Figure 5**), but also which intension of the term *artwork* they applied when they selected the stimuli. Furthermore, studies would benefit from an explanation on why the respective intensions were applied. Only then, the reader of the study will be fully informed.

Aesthetics research is very complex. Every single study faces different challenges associated with the usage of the term *artwork*. Therefore, it is impossible to provide the reader with exact instructions for specific experiments. Instead, the illustration of the ontological state, the given definitions of the intensions of the term *artwork*, the reference to the PIA Model and the decision tree are meant to assist future researchers to find their own way to internally valid (within one study) and externally valid (between studies) research.

## Limitations

This article provides a guideline for the handling of stimuli in empirical aesthetics research. However, due to its theoretical nature, it remains to be established whether the guideline will lead to more valid results. Possibly, a prospective meta-study on coherence and comparability could provide evidence whether empirical aesthetics truly benefits from the decision tree proposed in the present work. In such a study, aesthetic research that does not follow this guideline should be compared with aesthetic research that does follow this guideline. A success would be if research following this guideline leads to more conclusive and predictive results.

## Summary

Many studies in the field of empirical aesthetics struggle with the term *artwork*. Here, I argue that a lack of a clear definition may cause problems that stem mainly from the ontological state of artworks, as well as from a discrepancy between the selection and the usage of the experimental stimuli. As a guideline, a decision tree was created. If researchers follow the implications of this decision tree, future results may be more coherent and better comparable.

## Author Contributions

The author confirms being the sole contributor of this work and approved it for publication.

## Conflict of Interest Statement

The author declares that the research was conducted in the absence of any commercial or financial relationships that could be construed as a potential conflict of interest.
